# Promoting healthy sleep in Chinese kindergarteners through a family-based intervention: protocol of the “Healthy Sleep” randomised controlled trial

**DOI:** 10.1186/s12889-023-16806-1

**Published:** 2023-09-26

**Authors:** Zhiguang Zhang, Lin Li, Xiaohua Li, Anthony Okely

**Affiliations:** 1 Faculty of Education, Beijing Normal University, Beijing, 100875 China; 2 School of Early Childhood Education, Changsha Normal University, Changsha, 410100 China; 3 School of Health and Society, Faculty of Art, Social Sciences and Humanities, University of Wollongong, Wollongong, NSW Australia

**Keywords:** Movement behaviours, Cognitive development, Motor development, Social development, Pre-schooler

## Abstract

**Background:**

Sleep is instrumental for growth and development in children, making it critical to establish healthy sleep habits from the earliest years of life. Many kindergarteners (3–6 years) in China have inadequate and poor sleep, necessitating targeted interventions. This research protocol details the “Healthy Sleep” intervention that was designed to promote healthy sleep among kindergarteners in China.

**Methods:**

The “Healthy Sleep” intervention will be family-based and will support parents as change agents. The development of the intervention is based on evidence regarding correlates of sleep in young children and guided by Bandura’s social cognitive theory. A 12-month randomised controlled trial will be conducted to examine the efficacy of the intervention for promoting healthy sleep in Chinese kindergarteners and the intervention's effects on child development outcomes. A targeted sample of 160 kindergarteners and their parents will be recruited through social media. The intervention group (*n* = 80) will receive monthly webinars for one year that include multiple intervention components – including educational training, goal setting and planning, as well as follow-up support sessions. The control group (*n* = 80) will receive videos of the recorded educational sessions after the study end. For primary outcomes, child sleep behaviours will be examined using the Child Sleep Health Questionnaire. For secondary outcomes, communication, fine motor, gross motor, personal-social, and problem-solving development will be examined using the Ages and Stages Questionnaire; executive functions will be examined using the Head, Toes, Knees, and Shoulders Revised tasks. Potential intervention mediators and covariates will be measured using a parental questionnaire. Mixed models will be conducted.

**Discussion:**

This intervention targets sleep behaviours among kindergarteners in China. It has the potential to inform programs to support parents in helping their child establish healthy sleep habits from the earliest years of life. The study will provide high-quality experimental evidence on sleep behaviours in relation to development outcomes in kindergarteners. This evidence will inform family-based strategies to optimise early childhood development and inform national and international updates of the sleep recommendations for young children.

**Trial registration:**

The trial was registered prospectively at Chinese Clinical Trial Registry (ID: ChiCTR2300072105) on 2 June 2023.

**Supplementary Information:**

The online version contains supplementary material available at 10.1186/s12889-023-16806-1.

## Background

Early childhood is marked by significant physical, social, and cognitive development that lays the foundation for life-long well-being and achievements [1]. In the first few years of life, brain architecture and neuronal connections construct most actively, which forms a neural basis of cognitive function [2]. The pre-school years, for instance, is a period of rapid development of executive functions (i.e., a higher order of cognitive skills), such as working memory, inhibitory control, and cognitive flexibility [3]. Brain growth, along with the strengthening of muscles and the lowering of the centre of gravity, enable remarkable advances in the development of gross and fine motor skills during this period [2]. Additionally, important emotional and social growth, including acquisition of self-concept, social initiative, and empathy, occurs in the pre-school years [2]. These cognitive, motor, and social skills that emerge and develop in early childhood are associated with a number of key later-life outcomes, such as school readiness, academic performance, as well as physical and mental health [4, 5]. Therefore, it is important to promote healthy behaviours that support optimal early childhood development.

Healthy sleep is important for early childhood development. Evidence from observational studies has suggested that sleep is associated with adiposity levels [6, 7], motor skills [8], cognitive skills [7, 8], and social-emotional skills [9] in young children. Based on the best available evidence, the World Health Organisation (WHO) released guidelines that provide sleep duration recommendations for different age groups among children under five years of age [10]. In China, children typically attend kindergartens between the age of three to six years. To provide guidance for this age group, the Chinese movement behaviour guidelines for kindergarteners were specifically developed [11]. In line with the WHO guidelines for pre-schoolers [10], the Chinese guidelines recommend kindergarteners to sleep for 10 to 13 h per day. However, most Chinese kindergarteners may have inadequate sleep. A cross-sectional study examined total sleep duration using accelerometers in a sample of 254 kindergarteners in China and found that 72% of the children did not meet the sleep recommendation [12]. Apart from the quantitative aspect, sleep quality in many Chinese kindergarteners appears to be poor. A recent meta-analysis based on 27 Chinese studies with children aged 3 to 6 years revealed that 39% of the children had sleep problems, such as frequent night awakenings and trouble falling asleep [13]. These worrying statistics highlight the importance of promoting healthy sleep among kindergarteners in China.

Kindergarteners’ sleep behaviours occurs primarily at home and tends to be influenced by parents. A systematic review on correlates of sleep duration in early childhood suggests that multiple factors within the family context, including parental knowledge (e.g., health literacy level), parenting practices (e.g., bedtime routine), parental modeling (e.g., parental bedtime), parental emotion (e.g., stress), and home physical environment (e.g., bedroom television), are associated with child sleep duration [14]. Parents also play a role in managing child-level correlates of sleep duration, such as mood, screen use, and food intake [14]. In this sleep correlates systematic review, no studies examined parental attitude toward sleep [14]. This factor may be an important antecedent of parenting practices and potentially affect child sleep behaviours, particularly in the Chinese cultural context. Due to the influence of Confusion philosophy that emphasises academic success, Chinese parents often prioritise academic activities and place less value on healthy sleep [15]. Encouraging a shift in such parental attitudes by informing parents of the developmental benefits of healthy sleep, particularly in the cognitive domains that link with academic achievements, may motivate them to help their child establish healthy sleep habits. Therefore, a family-based intervention that addresses these sleep correlates within the family context and supports parents as change agents offers great potential to promote healthy sleep among kindergarteners. A recent systematic review and meta-analysis based on 31 studies showed that family-based interventions were efficacious to increase nighttime sleep duration in young children. However, the majority of the interventions (*n* = 26) targeted infants and toddlers [16]. Research focused on pre-school aged children is warranted to provide evidence-based strategies for promoting healthy sleep in this specific age group. Moreover, few sleep interventions were conducted in low- and middle-income countries and none in China [16]. Developing and testing a family-based sleep intervention targeting Chinese kindergarteners can provide a better understanding of effective strategies within a non-western cultural context.

While a growing body of research has examined sleep in relation to developmental outcomes in young children, most research used observational research designs that prevent causality inferences for the relationships. In a systematic review that informed the development of the WHO sleep recommendations, 64 out of 69 studies examining sleep duration in relation to health outcomes in children under the age of 5 years used observational research designs [17]. While the four remaining studies were experimentally designed, they focused on examining the impacts of acute sleep changes on cognitive and emotional regulation skills in infants and toddlers, with small sample sizes ranging from 7 to 23 participants [17]. It is unclear how habitual sleep influence developmental outcomes in young children, which is warranted to be examined in experimental studies with extended periods of intervention.

## Objective and hypothesis

The primary objective of this study is to examine the efficacy of a family-based intervention for promoting healthy sleep among kindergarteners in China. We hypothesise that there will be a greater increase in the proportion of children adhering to the sleep duration recommendation in the intervention group, compared to that in the control group. In addition, children in the intervention group will have greater improvement in sleep quality compared to those in the control group. The secondary objective is to examine the intervention's effects on child development outcomes.

## Methods

### Trial design

**Fig. 1:**
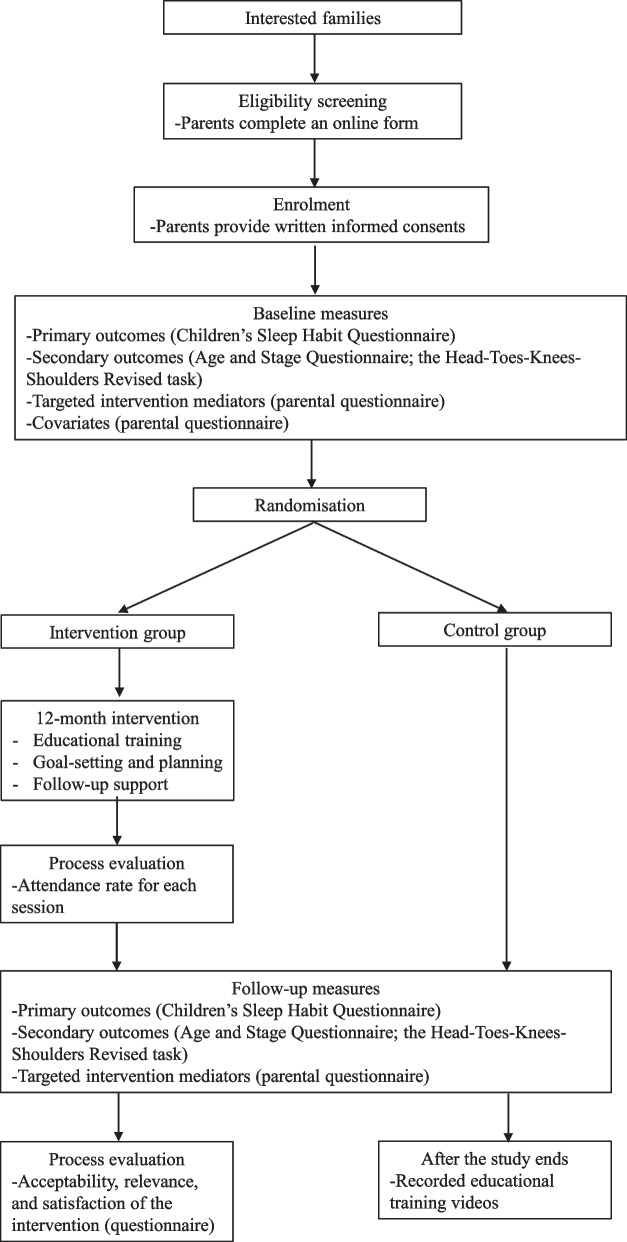
Flow diagram of the randomised controlled trial

The efficacy of “Healthy Sleep” intervention for improving sleep in kindergarteners will be evaluated using a 12-month 2-arm parallel group randomised controlled trial (RCT; Fig. 1). The trial was registered prospectively at Chinese Clinical Trial Registry (ID: ChiCTR2300072105) on 2 June 2023.

### Study setting

This family-based intervention will be conducted online, utilising a video conferencing platform—Tencent Meeting as the primary setting for intervention activities. This platform provides visual and audio communication channels, with screen-sharing capabilities, which enables real-time presentations and interactions. The online delivery method can provide flexibility and convenience to families who may have limited access to traditional face-to-face interventions due to geographical or time constraints.

### Eligibility criteria

Families will be eligible for this study if they: 1) have a child aged 3 to 6 years at baseline; 2) reside in mainland China; and 3) have a computer, laptop, tablet, or smartphone that can access the internet. Exclusion criteria for this study will include 1) children with medical or psychological conditions that may affect sleep or development, a diagnosed sleep disorder, or diagnosed developmental delays, and 2) children who have enrolled in a primary school.

### Intervention

The intervention will be grounded on Bandura’s social cognitive theory (SCT) that provides a robust theoretical framework for understanding behaviour change [18, 19]. The central construct of SCT is reciprocal determinism, which posits that individuals initiate and maintain a behaviour in a social context with a dynamic interaction between cognitive, behavioural, and environmental factors [18]. Specific to this intervention, the cognitive factors include parents’ beliefs and attitudes regarding healthy sleep, their knowledge of sleep needs for young children, and their self-efficacy in helping children establish and maintain healthy sleep habits. Behavioural factors include sleep-related parenting practices (e.g., establishing regular sleep schedule, implementing consistent bedtime routine, avoiding caffeine, and stimulating activities before bedtime) and child behaviours (e.g., self-soothing skills). Environmental factors include the physical characteristics of child sleep environment (e.g., the comfort of bed, noise levels, lighting, temperature), family and cultural norms regarding sleep, and parent modelling. The intervention will address these cognitive, behavioural, and environmental factors, while incorporating other key SCT constructs, including outcome expectancies, self-efficacy, observational learning, reinforcements, and self-regulation, into the intervention design and implementation [18, 19]. To guide the selection and implementation of evidence-based behaviour change strategies, this intervention will utilise the taxonomy of behaviour change techniques (BCTs) [20]. To enhance the relevance of the intervention, pre-intervention interviews will be conducted with potential participants of the study. The interviews will gather information regarding parental attitudes and beliefs regarding child sleep behaviours, awareness of the sleep recommendation, current sleep-related parenting practices, as well as barriers and facilitators they encountered when implementing sleep hygiene practices for their child. Parents will be individually interviewed via Tencent Meeting and their responses will be used to refine the intervention content.

The intervention will include multiple components aligning with specific SCT constructs (Table 1). Participants will attend 12 monthly 45-min webinars. The webinars (Webinar A) scheduled for months 1, 3, 5, 7, 9, and 11 include a 30-min educational training session and a 15-min goal setting and planning session. The educational sessions will be made up of six modules, guiding parents to address the cognitive, behavioural, and environmental factors related to child sleep behaviours via informative presentations (Table 2). This session will inform parents about the significance of healthy sleep in child development and provide them with evidence-based strategies to promote healthy sleep. The content of the educational session will be informed by the WHO guidelines [10] and the systematic review on the correlates of sleep in early childhood [14]. In the goal setting and planning session, parents will be encouraged to set challenging yet attainable goals that are related to their child’s sleep habits, which can help parents develop a sense of commitment and achievement towards promoting healthy sleep for their child. In this session, parents will receive guidance on creating personalised action plans that allow them to translate their goals into actionable steps, which will facilitate parents in implementing the learned strategies through a self-regulation process. The follow-up support sessions are included in the webinars (Webinar B) scheduled for months 2, 4, 6, 8, 10, and 12, allowing parents to engage in a group discussion regarding barriers and facilitators they encountered during the implementation phase. Through sharing their own experiences, receiving support from the research team, and gaining insights from their peers, parents will have an opportunity to reflect on their progress, adapt their strategies as needed, and troubleshoot encountered barriers.

**Table 1:** Intervention content

Intervention Components	Dose	Description	Behaviour change technique^1^	Social cognitive theory constructs
1. Educational training (Webinar A, session 1)	Bimonthly 30-min webinars (six in total at month 1, 3, 5, 7, 9, 11)	Parents will be educated about the importance of healthy sleep for child well-being and development, as well as the potential consequences of inadequate sleep. They will be introduced to evidence-based parenting practices for promoting healthy child sleep, such as establishing a consistent bedtime routine and creating a sleep-friendly environment. They will also be provided with practical guidance on implementing positive reinforcement strategies, such as using praise, encouragement, and rewards, to help their child develop and maintain healthy sleep habits	4.1 Instruction on how to perform a behaviour 4.2 Information about antecedents 5.1 Information about health consequences 5.6 Information about emotional consequences 6.1 Demonstration of the behaviour 7.1 Prompts/cues 8.1 Behavioural practice/rehearsal 8.3 Habit Formation 9.1 Credible source 9.2 Comparative imagining of future outcomes 10.3 Non-specific reward 10.4 Social reward 10.5 Non-specific incentive 11.2 Reduce negative emotions 12.1 Restructuring the physical environment 13.1 Identification of self as role model	- Reciprocal determinism -Outcome expectancies -Reinforcement -Observational learning
2. Goal setting and planning (Webinar A, session 2)	Bimonthly 15-min webinars (six in total at month 1, 3, 5, 7, 9, 11)	After each educational training session, parents will be encouraged to set specific goals related to their child’s sleep habits and create personalised action plans to implement the recommended parenting practices	1.1 Goal setting (behaviour) 1.4 Action planning	-Self-regulation -Self-efficacy
3. Follow-up support (Webinar B)	Bimonthly 45-min sessions (six in total at month 2, 4, 6, 8, 10, 12)	In each webinar, parents will be encouraged to share their experiences, challenges, and success related to the implementation of strategies to promote healthy sleep for their child. The research team will provide positive feedback and encouragement as well as will work with parents to brainstorm possible solutions to overcome the barriers	1.2 Problem solving 3.1 Social support (unspecified) 10.4 Social reward 15.1 Verbal persuasion about capability	-Self-efficacy -Self-regulation -Reinforcement -Observational learning

^1^These techniques were selected from BCT Taxonomy (v1) of 93 hierarchically-clustered techniques [﻿^20^]

**Table 2:** Interactive webinar themes

	**Themes**	**Content**	**Behaviour change technique**^**1**^
1	Sleep and healthy development	-The importance of promoting healthy sleep for child development, well-being, and academic achievement -Sleep need for young children	4.1 Instruction on how to perform a behaviour 5.1 Information about health consequences 5.6 Information about emotional consequences 9.1 Credible source 9.2 Comparative imagining of future outcomes
2	Relaxation for sleep	-Mood and sleep -Sleep relaxation techniques (e.g., deep breathing, progressive muscle relaxation, meditation, visualisation, soft music) -Avoiding screens before bed	4.2 Information about antecedents 11.2 Reduce negative emotions
3	Bedtime routine	-Benefits of a bedtime routine -Making the bedtime routine consistent -Recommended activities (e.g., reading books, storytelling, brushing teeth, bathing, changing pyjama, cuddling)	4.2 Information about antecedents 8.1 Behavioural practice/rehearsal 8.3 Habit Formation
4	Daily schedule	-Helping the child stick to a regular sleep schedule -Including physical activity in the child’s daily routine -Setting limits on the child’s screen time	4.2 Information about antecedents
5	Eating for sleep	-Effect of diet on sleep -Maintaining a balanced diet -Food and beverage to avoid before bedtime	4.2 Information about antecedents
6	A restful sleep environment	-Decluttering the sleep environment -Making the bed comfortable -Keeping the sleep environment cool, dark, and quiet -No screen devices in the sleep environment	4.2 Information about antecedents 12.1 Restructuring the physical environment 7.1 Prompts/cues

^1^These techniques were selected from BCT Taxonomy (v1) of 93 hierarchically-clustered techniques [20﻿]

### Recruitment

Recruitment will be primarily through social media that are popularly used in mainland China, such as WeChat, Weibo, and RED. Preschools will be contacted to help distribute information about this study to parents. In addition, a snowball recruitment strategy will be implemented. Parents or guardians (parents thereafter) who express interest in participating in this study will be requested to complete an online eligibility screening form before providing their consent to participate.

### Randomisation, allocation, and blinding

Data will be collected at baseline and follow-up (12-month post-baseline). After the baseline assessment, participants will be allocated randomly to the intervention or control groups on a 1:1 ratio by a research assistant not associated with the project. The control group will receive the videos of the educational training sessions after the study ends. The random allocation sequence will be computer generated using SPSS version 26.0 (SPSS Inc., Chicago, IL, USA). Allocation will be blinded for data collectors and participants.

### Measures

At each time point, data will be collected online through the Sojump platform, an internet-based survey application that is widely used in mainland China, and Tencent Meeting. Participating families will be provided with child assessment feedback to promote retention.

#### Primary outcome

The primary outcome of interest, pre-schoolers’ sleep behaviours, will be measured using the Chinese version of the Children’s Sleep Habit Questionnaire (CSHQ) [21, 22]. The CSHQ is a parental-report instrument, consisting of 33 items for scoring and several extra items intended to provide useful information about the child’s sleep behaviours [21]. The 33 items are grouped into eight subscales, measuring different aspect of sleep including: 1) bedtime resistance, 2) sleep-onset delay, 3) sleep duration, 4) sleep anxiety, 5) night wakings, 6) parasomnias, 7) sleep-disordered breathing, and 8) daytime sleepiness [21]. This instrument has shown to be reliable (Cronbach’s alpha: 0.68 to 0.78; test–retest reliability: 0.62 to 0.79) and valid (sensitivity = 0.80, and specificity = 0.72) for use in children aged 4 to 10 years [21]. A previous study further validated this instrument in children aged 2 to 5.5 years and observed significant correlations with small to large effect size between CSHQ and actigraph measures of sleep onset time (*r* = 0.48), night waking duration (*r* = 0.21), total 24-h sleep duration (*r* = 0.25), and morning awake time (*r* = 0.62) [23]. The Chinese version of the CSHQ has demonstrated good reliability (overall Cronbach’s alpha = 0.73; overall test–retest reliability = 0.85) for measuring child’s sleep [22].

#### Secondary outcomes

Child development will be measured using the Chinese version of the Ages and Stages Questionnaire—third version (ASQ-3) [24]. ASQ-3 is a developmental screening instrument for children aged 1–66 months and can be administrated by parents [24]. The instrument assesses five developmental domains, including communication, fine motor, gross motor, personal-social, and problem-solving areas. At each time point, each developmental domain will be assessed by six age-specific items. For each item, parents will be instructed to answer “yes”, “sometimes” or “not yet”, and following ASQ-3 procedures [24], the responses will be scored as 10, 5, and 0, respectively. Item scores will be summed to calculate a total sub-score ranging from 0 to 60. The five sub-scores will be totaled to calculate an overall score, ranging from 0 to 300, and higher scores indicate more advanced development. ASQ-3 has been validated against a few professionally administered standardized assessment [24]. Specifically, when compared to the Battelle Developmental Inventory-II, high percent agreements (74%-100%) were observed for the 36-month, 42-month, 48-month, 54-month, and 60-months questionnaires [24].

Children’s executive function will be measured objectively using the Head-Toes-Knees-Shoulders Revised (HTKS-R) task, which is available in the Chinese language [25]. The task is short (around 5–7 min to complete) and requires no special material to administer. It includes four parts and each part includes practice and test items. In part 1, children are asked to say the opposite of the instruction (e.g., “When I say toes, you say head”). In part 2, children are required to do the opposite of what is instructed for a single pair of commands (e.g., “When I say touch your head, you touch your toes” and vice versa). In part 3, another pair of commands is added (e.g., “When I say touch your knees, you touch your shoulders” and vice versa). In part 4, the pairs of commands are switched (e.g., “when I say touch your head, you touch your knees” and vice versa; “when I say touch your toes, you touch your shoulders” and vice versa). The four parts include a total of 59 items, consisting of 22 practice items and 37 test items. For each item, a correct, self-corrected, and incorrect response is scored as 2, 1, and 0, respectively, resulting in a maximum total score of 118. To respond correctly, children need to integrate multiple executive function skills, including working memory (i.e., remembering the rules while listening to the command), inhibitory control (i.e., inhibiting the more natural response to the instruction in favour of the unnatural one), and cognitive flexibility (shifting responses as the rules alternate). A trained research assistant will administer this task with children, who will be accompanied by a parent, via the online conferencing platform. The HTKS has been psychometrically tested in diverse samples of children ages 3 through 6, demonstrating excellent inter-rater reliability (k = 0.9) [25, 26]. A recent study has examined the construction validity of the HTKS-R in pre-schoolers and kindergarteners [25]. The task score was correlated with other measures of working memory (the Auditory Working Memory subtest of the Woodcock-Johnson Tests of Achievement: *r* = 0.19–0.61), inhibitory control (the Day-Night Stroop task: *r* = 0.27–0.43), and cognitive flexibility (the Dimensional Change Card Sort task: *r* = 0.27–0.44) [25].

#### Potential intervention mediators

Targeted mediators of the intervention, including parental attitudes and beliefs towards child sleep behaviours, parental knowledge of the sleep recommendation, self-efficacy, parenting practices, parental modelling, and home environments, will be collected via a parental questionnaire.

#### Covariates

Data on covariates, including child date of birth, gender, number of siblings, and kindergarten attendance, parental year of birth, marital status, and education, household income, grandparent involvement, as well as geographic regions, will be collected via the parental questionnaire.

#### Process evaluation

Fidelity of the intervention will be assessed as attendance rate for each webinar. At the 12-month follow-up, parents in the intervention group will be asked about the acceptability, relevance, and satisfaction of the intervention via a questionnaire.

### Sample size

A sample size calculation was conducted based on repeated MANOVA (two groups, two time points) using G*Power (version 3.1.9.7). To detect a medium effect size (f = 0.25) [27] for the between-groups differences in changes in the primary outcomes, with a power of 0.8 and a Type I error probability of 0.05, the required sample size was calculated to be 128 participants. To further accounted for 20% loss to follow-up, a minimum sample size of 160 participants will be recruited.

### Statistical analysis

To address the primary objective and the secondary objective, a two-level (i.e., level 1 = time, level 2 = subject) model will be employed to examine the intervention effect on each outcome. The model for each outcome will include a random effect of subject and fixed effects of time, group, time*group, and covariates. The beta coefficient for the interaction term “time*group” is interpreted as the difference in changes in the outcome, from 12-month follow-up to baseline timepoints, between intervention and control groups, indicating the intervention effect. To further examine the intervention pathways, multilevel mediation analysis will be used to examine the indirect effects of the intervention on child sleep behaviours through the targeted mediators. Data collected from the pre-intervention interviews will be transcribed verbatim and analysed thematically.

### Ethics, data management, and dissemination

Ethical approval for the study was obtained from the Beijing Normal University Faculty of Education Research Ethics Committee (IRB Number: BNU202212100058). After eligibility screening, parents will be required to provide written informed consent for their own and their child’s participation in the study. Participants will be informed that their participation is voluntary and they can withdraw without penalty at any time. The likelihood and extent of potential harm resulting from participation are not anticipated to exceed those encountered in participants’ daily lives related to the study. To minimize the burden on participating families, we will not collect data on adverse events. Identifying information will be collected through the consent form and each participant will be assigned a unique participant number. Collected data will be linked to participant numbers, not identifying information. Data will be stored on password-protected computers, with access limited to the research team. A master file containing participant numbers and identifying information will be destroyed securely at the conclusion of data collection. Deidentified data will be retained for a minimum of ten years after study completion. The study findings will be disseminated through peer-reviewed publications, presentations at academic and professional conferences, as well as social and traditional medias. These knowledge translation activities will be of general findings and will not breach individual confidentiality.

## Discussion

Early childhood is a period when sleep patterns evolved most rapidly [28]. Sleep patterns during the pre-school period are relatively more stable compared to infancy and toddlerhood but continue to change [28]. Unhealthy sleep patterns formed in the early years tend to track into later childhood and have long-lasting effects on health and development [14]. Inadequate and poor sleep were observed in a large proportion of kindergarteners in China [12, 13], warranting interventions targeting this population. However, in a recent published systematic review on sleep interventions for young children, no studies have been conducted in China [16]. The study described in the present protocol will be, to our best knowledge, the first intervention promoting healthy sleep among kindergarteners in China and is developed with an evidence-based and theory-driven approach. This intervention will be centred around family units, given evidence on the important role of family settings and parental factors in shaping young children’s sleep behaviours [29]. The design of intervention components is guided by the SCT to support parents as the change agent [18, 19]. To increase relevance, the content of the intervention is tailored to cultural contexts and will be refined according to the pre-intervention interviews. Findings from this intervention will provide important insights on effective strategies for parents, especially those from Chinese cultural backgrounds, to help their child establish healthy sleep habits. Moreover, this intervention is online-based and, if efficacious, has the potential to be offered widely to parents, including those residing in rural and remote areas, to support them in promoting healthy sleep for their child from the earliest years of life.

This study will use a rigorous research design to examine the intervention effects on development outcomes and the mediating role of sleep. The findings can provide high-quality evidence on sleep in relation to development outcomes in kindergarteners from low- and middle-income countries. This evidence will be important to inform the international and national updates of the sleep recommendation as the development of current sleep recommendations was based on evidence that relied heavily on observational studies conducted in high-income countries [17]. Findings from this study also have the potential to inform effective strategies and programs to support healthy development in children from Chinese cultural backgrounds.

### Supplementary Information

**Additional file 1.** The SPIRIT Checklist.

## Data Availability

Not applicable.

## Abbreviations

ASQ-3: Ages and Stages Questionnaire – third version
BCT: Behaviour Change Technique
CSHQ: Children’s Sleep Habit Questionnaire
HTKS-R: Head-Toes-Knees-Shoulders Revised
RCT: Randomised Controlled Trial
SCT: Social Cognitive Theory
WHO: World Health Organisation
